# Association of prenatal alcohol exposure with offspring DNA methylation in mammals: a systematic review of the evidence

**DOI:** 10.1186/s13148-022-01231-9

**Published:** 2022-01-21

**Authors:** Mitchell Bestry, Martyn Symons, Alexander Larcombe, Evelyne Muggli, Jeffrey M. Craig, Delyse Hutchinson, Jane Halliday, David Martino

**Affiliations:** 1 Telethon Kids Institute, The University of Western Australia, Perth, WA Australia; 2 National Drug Research Institute, Faculty of Health Sciences, Curtin University, Perth, Australia; 3 Wal-yan Respiratory Research Centre, Telethon Kids Institute, Perth, Australia; 4 School of Population Health, Curtin University, Perth, Australia; 5 Victorian Infant Brain Studies, Murdoch Children’s Research Institute, Parkville, VIC Australia; 6 Department of Paediatrics, The University of Melbourne, Parkville, VIC Australia; 7 Murdoch Children’s Research Institute, Parkville, VIC Australia; 8 IMPACT – the Institute for Mental and Physical Health and Clinical Translation, School of Medicine, Deakin University, Geelong, Australia; 9 Public Health Genetics, Murdoch Children’s Research Institute, Parkville, VIC Australia; 10 Centre for Social and Early Emotional Development and Centre for Drug, Alcohol and Addiction Research, School of Psychology, Faculty of Health, Deakin University, Geelong, Australia; 11 Centre for Adolescent Health, Royal Children’s Hospital, Melbourne, Australia; 12 National Drug and Alcohol Research Centre, University of New South Wales, Sydney, Australia

**Keywords:** DNA methylation, Epigenetics, Fetal alcohol spectrum disorder, Prenatal alcohol exposure, Pregnancy

## Abstract

**Background:**

Prenatal alcohol exposure (PAE) is associated with a range of adverse offspring neurodevelopmental outcomes. Several studies suggest that PAE modifies DNA methylation in offspring cells and tissues, providing evidence for a potential mechanistic link to Fetal Alcohol Spectrum Disorder (FASD). We systematically reviewed existing evidence on the extent to which maternal alcohol use during pregnancy is associated with offspring DNA methylation.

**Methods:**

A systematic literature search was conducted across five online databases according to Preferred Reporting Items for Systematic Reviews and Meta-Analyses (PRISMA) guidelines. PubMed, Web of Science, EMBASE, Google Scholar and CINAHL Databases were searched for articles relating to PAE in placental mammals. Data were extracted from each study and the Risk of Bias in Non-Randomized Studies of Interventions (ROBINS-I) was used to assess the potential for bias in human studies.

**Results:**

Forty-three articles were identified for inclusion. Twenty-six animal studies and 16 human studies measured offspring DNA methylation in various tissues using candidate gene analysis, methylome-wide association studies (MWAS), or total nuclear DNA methylation content. PAE dose and timing varied between studies. Risk of bias was deemed high in nearly all human studies. There was insufficient evidence in human and animal studies to support global disruption of DNA methylation from PAE. Inconclusive evidence was found for hypomethylation at IGF2/H19 regions within somatic tissues. MWAS assessing PAE effects on offspring DNA methylation showed inconsistent evidence. There was some consistency in the relatively small number of MWAS conducted in populations with FASD. Meta-analyses could not be conducted due to significant heterogeneity between studies.

**Conclusion:**

Considering heterogeneity in study design and potential for bias, evidence for an association between PAE and offspring DNA methylation was inconclusive. Some reproducible associations were observed in populations with FASD although the limited number of these studies warrants further research.

*Trail Registration*: This review is registered with PROSPERO (registration number: CRD42020167686).

**Supplementary Information:**

The online version contains supplementary material available at 10.1186/s13148-022-01231-9.

## Background

Alcohol use during pregnancy is a preventable cause of offspring neurodevelopmental impairments. Alcohol exposure in utero has been associated with birth defects [1], as it passes freely across the placenta, and unlike adults, the exposed fetus has minimal ability to metabolise alcohol. The timing, dose and frequency of consumption is associated with the severity of prenatal alcohol exposure (PAE) on fetal development [2]. PAE can cause Fetal Alcohol Spectrum Disorder (FASD), a diagnostic term that encompasses a spectrum of physical, cognitive, behavioural and neurodevelopmental abnormalities with life-long health consequences [3]. FASD is a significant health burden globally affecting approximately 7.7 per 1000 individuals, with a significantly higher burden identified among specific disadvantaged and Indigenous communities, within corrective and psychiatric services, and children in foster care [4]. At moderate to low doses the evidence for harmful effects on child development is controversial [5–8]. PAE is necessary but not sufficient to cause FASD in all exposed individuals and research is needed to better understand disease etiology including the genetic and other environmental cofactors potentially influencing the clinical manifestation of FASD [9]. Improvements in screening and diagnosis are research priorities [10] in order to facilitate timely and appropriate interventions, and for this to occur a clearer understanding of mechanisms is required.

Recent evidence suggests epigenetic processes such as DNA methylation as a potential mediator of PAE on offspring neurodevelopment and FASD. Epigenetic processes are reversible structural and functional modifications to the genome that play an important role in the regulation of fetal gene expression. Evidence from both human [11–13] and animal studies [14–16] suggests that PAE and FASD are associated with changes to global and gene-specific levels of DNA methylation. The addition of methyl groups to cytosine residues on DNA can modify the structural density of DNA, and its compaction within the nucleus, with consequences for regulation of fetal gene expression. Ethanol is readily able to cross the placenta and accumulate in the fetal tissues at levels proportional to the maternal blood alcohol concentrations [17]. Studies suggest this can affect the patterning of DNA methylation through either direct inhibition of DNA methyltransferase enzymes, or through antagonist effects on dietary methyl donors such as folate and choline, which are substrates for these enzymes [18]. Evidence to date has been inconsistent, with multiple different associations reported in murine models and in human populations with clinical FASD [19]. This is in contrast to evidence from multiple birth cohorts which suggest low to moderate PAE does not affect offspring DNA methylation [20]. Potential confounding factors represent a major challenge since multiple dietary and environmental exposures in pregnancy also influence offspring DNA methylation outcomes. Due to these inconsistencies and limitations in the literature, we conducted a systematic review of the extant research in this field to synthesise current evidence for DNA methylation as a mediator of PAE. Considering the problem of residual confounding in observational studies, we synthesized evidence for an association between PAE and offspring DNA methylation outcomes in placental mammals by examining evidence from both human studies and controlled animal models.

## Methods

This systematic review was guided by the Preferred Reporting Items for Systematic reviews and Meta-Analyses (PRISMA) 2020 guidelines [21]. The protocol for this systematic review was registered with the International Prospective Register of Ongoing Systematic Reviews (PROSPERO) on 05 July 2020 (registration number: CRD42020167686) [22].

### Inclusion criteria

Studies of prenatal alcohol exposure in placental mammals, in which DNA methylation was measured as an outcome were considered for inclusion.

### Exclusion criteria

In vitro models or postnatal exposure studies were excluded as they do not reflect placental contribution to the exposure. Case reports, narrative reviews and studies without DNA methylation outcomes were also excluded.

### Types of studies and participants

The review included cross-sectional studies, longitudinal cohorts and randomised control studies in humans and animals. Participants were placental mammals with confirmed PAE or diagnosis of FASD. Non-mammals, solely paternal, postnatal, or pre-conception exposure studies were excluded along with case reports and in vitro only studies.

### Interventions and comparisons

The primary intervention among included studies was in utero exposure to ethanol, by any delivered method and dose, for any duration. To be eligible, studies were required to have a comparison group with no alcohol exposure at any time during pregnancy. We also included studies reporting on cohorts with a confirmed diagnosis of FASD with a comparison group of typically developing controls.

### Outcome measures and timing

Studies were required to provide quantitative offspring DNA methylation outcomes, either in a CpG or non-CpG context. DNA methylation was able to be reported across the entire methylome or within a specific locus or loci. The main outcome assessed was differences in DNA methylation between the PAE case and non-PAE control groups, as indicated by use of a statistical test assessing differences in DNA methylation between the case and control groups.

Any tissue or cell type extracted from the mammal was considered. We also considered whether changes in the DNA methylation from PAE were dependent on the tissue or cell type.

#### Search strategy

A literature search was conducted on 30 January 2020 to identify relevant articles across five databases: PubMed, Web of Science, EMBASE, Google Scholar and CINAHL Plus. The keyword search terms used for each database were: methylat* and (alcohol* or ethanol*) and (foetal* or fetal* or prenatal* or antenatal* or perinatal* or pregnan* or maternal* or utero* or foetus* or fetus* or intrauterine* or alcohol-related birth disorder* or alcohol-related neurodevelopmental disorder*). Only English language articles were included in this review. There was no restriction on the publication period.

The literature search was repeated on 17 July 2021 across the same databases to identify any additional articles that were published during the time period.

#### Synthesis of the data

References were imported into EndNote software to remove duplicates, non-original research articles and articles that were not in English. Titles and abstracts were double screened independently by two authors (MB and MS) using Covidence software. Disagreements were resolved through discussion with a third screener (DM). Following the same procedure as the title and abstract screening, Covidence was again used for full-text screening.

Due to the small number of articles in the updated search, articles underwent title and abstract screening followed by full-text screening.

Data extraction was conducted by two researchers (MB and MS) using a pre-designed form adapted from the James Cook University Data Extraction Form (Additional file 1: Fig. 1). Data extracted from each study included: study design, population studied (i.e., species), methods for recruitment, sample size and demographics, cell types (including heterogeneity) that underwent DNA methylation analysis, pattern of PAE, DNA methylation assay used, reported candidate loci and, DNA methylation measures. Participant demographics included age, sex, ethnicity, smoking status, diet, socio-economic status, and rodent strain, where appropriate were extracted. Two researchers (MB and MS) each conducted 50% of the data extraction, after which they cross-checked the other’s data extraction. Discrepancies were resolved by consensus and reviewed by a third party (DM). Studies were grouped by species, cell or tissue type, whether they assessed the entire genome or only certain loci and method of DNA methylation analysis.

For human studies potential for bias was assessed using the Risk Of Bias In Non-randomized Studies of Interventions (ROBINS-I) approach by two researchers independently (DM and MS) with disagreements resolved through discussion [23].

## Results

### Search results

**Fig. 1:**
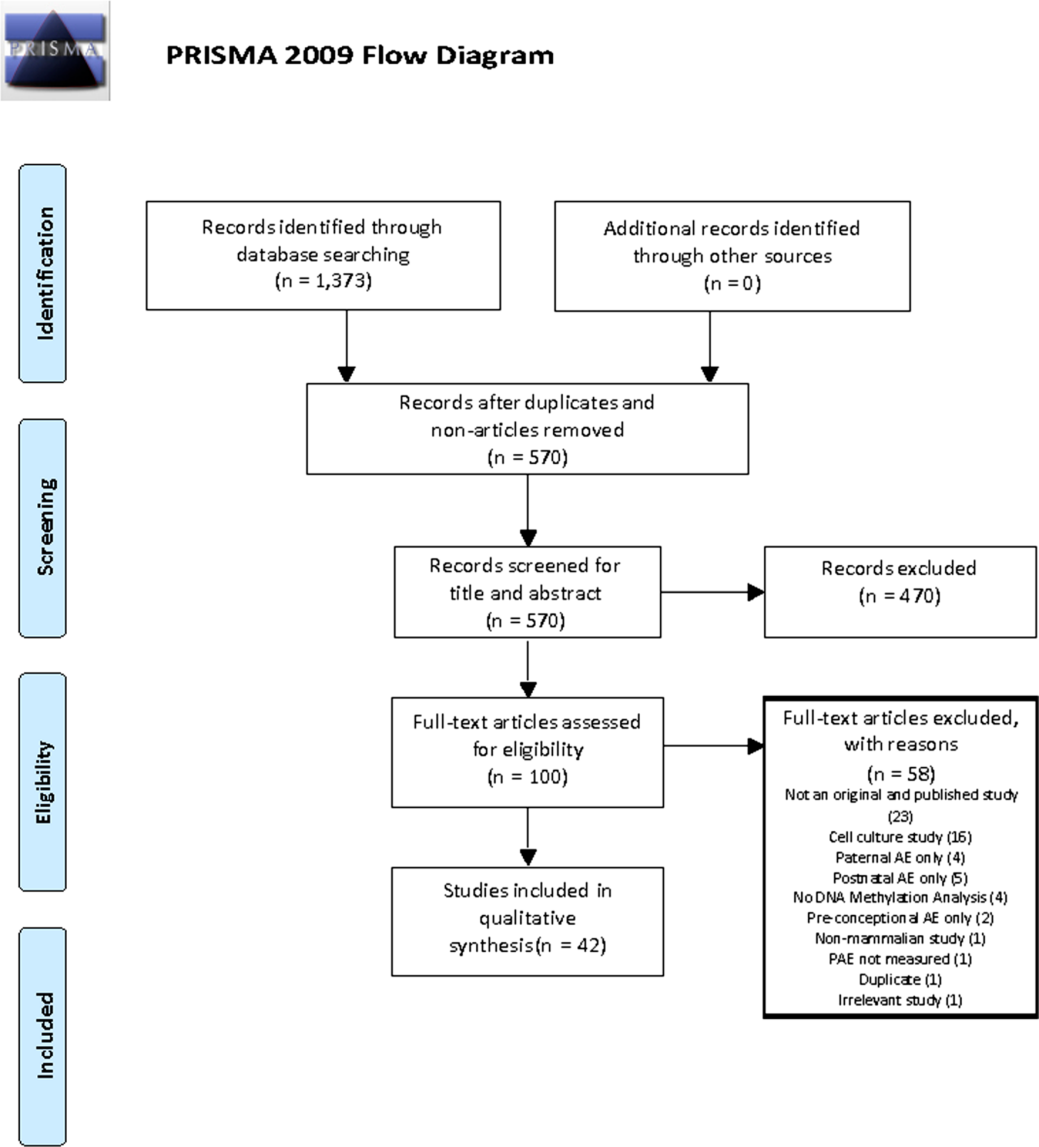
PRISMA flowchart outlining the study selection process across both searches

Figure 1 shows the PRISMA flowchart outlining the study selection process across both searches. A total of 1373 articles were obtained from the original and updated searches. The number of articles obtained by database were: PubMed (n = 254), Web of Science (n = 426), EMBASE (n = 543), Google Scholar (n = 106) and CINAHL Plus (n = 42). After removal of duplicates (n = 550), non-English articles, books, theses and conference abstracts, 442 references were imported into Covidence for title and abstract screening. A total of 344 articles were removed in the title and abstract screening phase, resulting in 98 articles that underwent full-text screening. A total of 58 articles were excluded during full-text screening (Fig. 1). A total of 42 articles were included in the review.

### Characteristics of included studies

Forty-two of the included articles were published between 1991 and 2020. There were 26 animal studies with approximately 1373 subjects, and 16 human studies with approximately 6788 participants. However, sample sizes are approximations as the information provided in some studies was unclear and only estimated from the articles. There were nine studies assessing total nuclear DNA methylation content, eleven MWAS and 24 studies that assessed DNA methylation within a specific locus or loci. This includes three studies that assessed total nuclear DNA methylation content and DNA methylation content within specific loci. Organism and number of participants, year published, study design, exposure pattern, tissues studied and DNA methylation analysis platform used are summarised in Table 1.

**Table 1:** Summary table of articles included in this systematic review

First author	Year	Organism	Study type	Scale	Loci (if targeted)	Total sample size	Exposure pattern	Ethanol exposure time (gestational days)	DNA methylation analysis platform	Tissues
Wille-Bille	2020	Wistar Rats	Intervention	Targeted	*Pdyn*, *Kor*	32	Moderate	GD 17–20	Pyrosequencing	Ventral Tegmental Area (PDYN), Amygdala (KOR)
Alberry	2020	C57Bl/6 Mouse	Intervention	MWAS	-	21	Moderate	GD 0–21	MeDIP-Chip	Hippocampus
Abbott	2018	CD1 Mouse	Transgenerational	Global + Targeted	*Id2* and *Rzrβ* promoters	195	Moderate-high	GD 0.5–19.5	MSP + ELISA	Rostral & Caudal regions of neocortex
Lussier	2018	Sprague–Dawley Rats	Intervention	MWAS	-	39	Moderate	GD 1–21	Bisulfite sequencing	Hippocampus, white blood cells
Marjonen	2018	C57Bl/6 J Mouse Rcc	Intervention	Targeted	*Igf2*/*H19* imprinting control region (ICR), *Igf2* DMR 1, *Snrpn* ICR, *Peg3* ICR	10	Moderate	GD 0.5–8.5	MassARRAY EpiTYPER	Embryonal + placental
Lu	2018	Wistar Rats	Intervention	Targeted	*Gad67*	16	Unclear, possibly high	GD 9–20	Amplicon sequencing	Hippocampus
Wille-Bille	2018	Mouse	Intervention	Targeted	*Pdyn*, *Kor*	64	Moderate	GD 17–20	MSP	Ventral Tegmental Area, NAc (PDYN only), Prefrontal Cortex (KOR only)
Ozturk	2017	C57Bl/6 Mouse	Intervention	Global	-	18	Moderate	GD 7–16	ELISA	Neocortex
Zhou	2016	C57Bl/6 J Mouse	Intervention	Targeted	Igf2/H19, DMR0, DMR1, DMR2, H19 DMR	33	High	GD 0.5–15.5	MassARRAY EpiTYPER	Heart, brain, placental
Zhang	2015	C57Bl/6 Mouse	Intervention	Targeted	From -144 to -20 bp *Slc17a6* transcription start site	109	Moderate	GD 0–8.5	Bisulfite sequencing	Hippocampus
Ngai	2015	Sprague–Dawley Rats	Intervention	Targeted	*Nr3c1* and *Slc6a4*	18	Unclear	GD 1-? (possibly 20)	Pyrosequencing	Hippocampus, hypothalamus
Marjonen	2015	C57Bl/6 J Mouse Rcc	Intervention	Targeted	Six CpG islands in each of *Olfr110*, *Vmn2r64*, *Vpreb2*, *Olfr601* and *Hist1h2ai*	18	Moderate	GD 0.5–8.5	Amplicon sequencing	Hippocampus, main olfactory epithelium
Gangisetty	2015	Fisher-344 Rats	Intervention	Targeted	*D2r* promoter	18	Moderate	GD 7–21	MSP + pyrosequencing	Pituatry gland
Gangisetty	2014	Sprague–Dawley Rats	Intervention	Targeted	*Pomc*	18	Moderate	GD 7–21	MSP	Mediobasal hypothalamus
Chen	2013	C57Bl/6 Mouse	Intervention	Global	-	17	Moderate-high	GD 7–16	Immunohistochemistry	Hippocampus
Laufer	2013	C57Bl/6 Mouse	Intervention	MWAS	-	12	Unclear	GD 0–10	MeDIP-Chip	Whole brain
Bekdash	2013	Sprague–Dawley Rats	Intervention	Targeted	*Pomc*	24	Moderate	GD 7–21	MSP	Mediobasal hypothalamus
Govorko	2012	Sprague–Dawley Rats	Transgenerational	Global + Targeted	*Pomc*	45	Moderate	GD 7–21	MSP + pyrosequencing + Immunohistochemistry	Brain
Downing	2011	C57BL/6 J (B6) Mouse	Intervention	Targeted	*Igf2* DMR1, four CpG sites	45	High	GD 0–8.5	Amplicon sequencing	Embryonal + placental
Stouder	2011	Normal FVB/N Mouse	Intervention	Targeted	*H19*, *Gtl2*, *Peg1*, *Srnpn*, *Peg3*	38	Light	GD 10–18	Pyrosequencing + Amplicon Sequencing	Tail, liver, tibialis anterior muscle, hippocampus, whole brain, sperm
Kaminen-Ahola	2010	C57Bl/6 Mouse	Intervention	Global + Targeted	A(vy) allele	373	Moderate	GD 0.5–8.5	Bisulfite sequencing	Forebrain, tail
Haycock	2009	C57Bl/6 & CastEi Hybrid Mouse	Intervention	Targeted	*Igf2*/*H19*	10	High	GD 1.5 & 2.5	Bisulfite sequencing	Embryonal + placental
Murillo-Fuentes	2005	Wistar Rats	Intervention	Global	-	32	Moderate-high	GD 0–8.5	Tritiated thymidine incorporation	Blood, liver
Maier	1999	Harlan Sprague–Dawley Rats	Intervention	Targeted	*Bdnf*	9	High	Unclear, possibly GD 1–20	Restriction digest + Southern Blot	Olfactory bulbs
Valles	1997	Wistar Rats	Intervention	Targeted	*Gfap*	4	Unclear, possibly moderate	Presumably GD 1–20	Restriction digest + Southern Blot	Brain
Garro	1991	Swiss-Webster Mice	Intervention	Global	-	164	High	GD 9–11.5	Tritiated thymidine incorporation	Fetal
Okazaki	2021	Human	Case–control study	MWAS	Five epigenetic clocks	12	FASD	-	Illumina 450 Beadchip	Buccal, peripheral blood
Timms	2019	Human	Observational Cohort Study	MWAS	-	783	Unclear	Unclear	Illumina 450 Beadchip	Cord blood
Cobben	2019	Human	Case–control study	MWAS	-	138	FASD	-	Illumina 450 Beadchip	Whole blood
Sarkar	2019	Human	Case–control Study	Targeted	*PER2*, *POMC*	224	Moderate-high	Month of conception and/or before birth (study 1), throughout pregnancy (study 2)	MSP + pyrosequencing	Buccal
Lussier	2018	Human	Case–control study	MWAS	-	48	FASD	-	Illumina 450 Beachchip + pyrosequencing	Buccal
Frey	2018	Human	Observational Cohort Study	MWAS	-	156	Unclear	Last 8 weeks of pregnancy	Illumina 450 Beadchip	Buccal
Sharp	2018	Human	Meta-analysis	MWAS	-	3075	Multiple patterns assessed (low/moderate and binge)	Second/third trimester	Illumina 450 Beadchip	Cord blood
Marjonen	2017	Human	Case–control study	Targeted	*IGF2*/*H19*	75	Moderate-high	Likely throughout	MassARRAY EpiTYPER	Placenta
Portales-Casamar	2016	Human	Case–control study	MWAS	-	206	FASD	-	Illumina 450 Beachchip + pyrosequencing	Buccal
Fransquet	2016	Human	Observational Cohort Study	Targeted	*DRD4* promoter	536	Multiple patterns assessed	Multiple patterns assessed	MassARRAY EpiTYPER	Buccal
Laufer	2015	Human	Case–control study	MWAS	-	23	FASD	-	Illumina 450 Beachchip + pyrosequencing	Buccal
Lee	2015	Human	Observational Cohort Study	Targeted	*DAT*, *SERT*, *MeCP2*	164	Multiple patterns assessed (light, moderate and binge)	Likely throughout	Restriction digest + MSP	Cord blood
Masemola	2015	Human	Case–control study	Targeted	*H19* ICR, *IG-DMR*, *KvDMR1*, *PEG3* DMR	533	FAS	Unclear/likely throughout	Pyrosequencing	Whole blood (all), buccal (cases only)
Azzi	2014	Human	Observational Cohort Study	Targeted	*ZAC1*	213	Unclear	Last 3 months of pregnancy	MSP	Cord blood, leukocytes
Jarmasz	2019	Human & Macaque Monkeys	Case–control study	Global	-	36	Variable	Variable	Immunohistochemistry	Brain
Loke	2018	Human	Observational Cohort Study	Global	-	187	Variable	Multiple patterns assessed	MassARRAY EpiTYPER	Placenta

The repeat of the literature searches on 17 July 2021 identified two new studies that were compliant with the inclusion and exclusion criteria of this review.

### Study design and participants

Human studies comprised 38% of the studies included in this review (16/42). Among the human studies there was substantial heterogeneity in study design (56% case–control studies, 38% observational studies, 6% meta-analytic studies); the tissue used (50% buccal swabs, 44% varying types of blood cells, including cord blood, 12% placental tissue, 6% brain tissue); and the platform used to measure DNA methylation outcomes (50% microarray, 31% sequencing, 19% mass spectrometry, 19% polymerase chain reaction, 12% enzyme digest/immunohistochemistry) (Table 1). Multiple platforms and tissue types were used in some studies. Most human studies were either targeted candidate gene analyses (6/16) or methylome-wide association studies (MWAS) of PAE (4/16) or FASD (4/16). The remaining two human studies used a global measure of total nuclear DNA methylation content.

All of the animal studies employed an intervention case–control design with all but one model being a rodent species (58% mouse, 42% rat) and a single model using non-human primates. The majority of animal studies measured DNA in brain tissue (21/26, 81%). Other tissues analysed included placenta, heart, tail, liver, blood and embryonal tissue. Most animal studies were targeted candidate gene analyses (19/26, 73%) and/or used a global measure of total nuclear DNA methylation content (7/26, 27%), with only three studies (12%) performed at methylome-wide scale. The dosage of PAE varied between animal studies including light (1/26, 4%), moderate (10/26, 38%), moderate-high (3/26, 12%), high (6/26, 23%), and unclear (6/26, 23%). The timing of PAE also varied in the animal studies including PAE across pregnancy (5/26, 19%), the first trimester only (6/26, 23%), the second trimester only (1/26, 4%), the third trimester only (2/26, 8%), an alternative PAE pattern (9/26, 35%), or an unclear timing (3/26, 12%).

### Risk of bias assessment

**Fig. 2:**
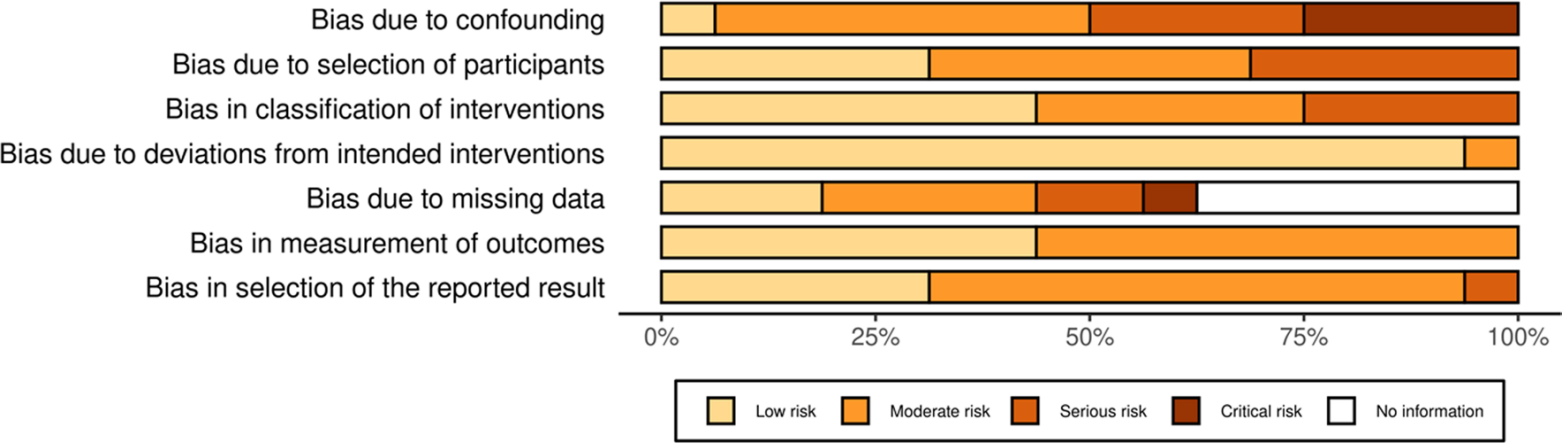
Summary chart of risk of bias assessment for human studies. The Percentage of studies scored for each bias domain is shown

Risk of bias was only assessed for the human studies since bias is inherently more problematic in these types of study designs than in controlled animal models. Bias due to baseline confounding or effect modification was a major issue as many studies did not consider or appropriately control for the effects of maternal smoking, diet, ethnicity or cellular heterogeneity, all of which can influence DNA methylation results (Fig. 2). Exposure groups were generally clearly defined, although usually determined through self-reporting, which may underestimate true exposure [24]. Many studies provided inadequate information on whether the same criteria were used to define cases and controls, or on missing outcome or covariate data (Fig. 2). Individual study bias scores are provided in Additional file 1: Fig. 1.

### Summary of outcomes

#### Global measures of DNA methylation

There were eleven studies that assessed total nuclear DNA methylation content including two human studies and nine animal models. Of the former, Loke et al. reported no association between PAE and total placental DNA methylation, but reported hypermethylation (1.5%, p = 0.012) in stratified analysis of males exposed to any level of alcohol throughout pregnancy, after adjusting for confounders [25]. Jarmasz et al. reported hypomethylation of nuclei from hippocampal CA1 region neurons (p = 0.02) in postmortem early fetal tissues (21–25 weeks post conception) with a history of alcohol abuse, although the analysis was not adjusted for multiple testing and no measure of effect size was given [11]. The authors did not reproduce this association in a non-human primate model of high dose exposure in CA1 neurons using the same techniques.

Among the animal studies, Chen et al. reported that moderate-high alcohol doses (4% ethanol, Gestational Day (GD) 7–16) given to C57Bl/6 dams were associated with alterations in DNA methylation during fetal hippocampus development [15]. Immunostaining for DNA methylation and DNA hydroxy methylation were significantly lower in the neuroepithelial layer, but higher in the intermediate zone and CA1 neurons in affected embryos. At postpartum day seven, methylation levels were higher in the outer shell layer of the dentate gyrus, whilst hydroxy methylation levels were lower in both the inner and outer shell layers. Öztürk et al. replicated the study and reported hypermethylation within CP subcortical regions in the exposed embryos, but no effect on other subcortical regions [26]. Abbott et al. reported global hypomethylation in neocortex samples in CD1 pups exposed to a high dose exposure regime across pregnancy (25% ethanol, GD0.5-GD19) [27]. Garro et al. also reported global DNA hypomethylation using a DNA methyl acceptor assay in unspecified fetal tissue from Swiss-Webster dams exposed to high levels of ethanol (3 g of 50% ethanol per kilogram body weight) by gavage in part of the second trimester of pregnancy (GD9-11) [28]. Govorko et al. found no evidence of global changes in DNA methylation in the arcuate nucleus brain region of offspring of Sprague–Dawley rats exposed to moderate levels of ethanol during gestation (variable 1.7–5.0% ethanol ad libitum within liquid diet during GD7-10 followed by 6.7% ethanol during GD11-21) [29]. Similarly, Kaminen-Ahola et al. also found no evidence of global changes to DNA methylation in tail or forebrain samples from C57Bl/6 offspring mice of dams exposed to moderate levels of alcohol for eight days after fertilization (10% ethanol, GD0.5–8.5) [30]. Murillo-Fuentes et al. also reported no difference in global DNA methylation in liver tissue among Wistar pups exposed to moderate-high doses across pregnancy (5–15% ethanol) and then maintained at 20% for a further 5 weeks during lactation [31].

Taken together, human and animal study evidence of an effect of PAE on overall nuclear level of DNA methylation was inconsistent, and no correlations between the timing and dose of exposure and methylation outcomes were identified.

#### Candidate gene studies

Candidate gene studies assessed DNA methylation at imprinted genes (*IGF2/H19* [13, 32–38], *Snrpn* [37], *PEG3* [35, 37], *KvDMR1* [13], *ZAC1* [39], *IG-DMR* [13]), genes sensitive to nutritional programming (A^vy^ allele [30], *Pomc* [29, 40, 41]), neurological function (*SLC17A6* [42], *OLFR110* [43], *OLFR601* [43], *VMN2R64*-*ps158* [43], *D2r* [44], *Bdnf* [45], *Pdyn* [46, 47], *Kor* [46, 47], *SLC6A4* [48], *Nr3c1* [48], *Gfap* [49], *Gad67* [14], *DRD4* [12], *PER2* [50], *SLC6A4* [48], *SERT* [51], *MECP2* [51]), transcriptional regulation (*Hist1h2ai* [43]), and immune function (*Vpreb2* [43]). Table 2 summarises all candidate gene studies in this review that assessed the Igf2/H19 locus,.

**Table 2:** Summary table of candidate gene studies assessing DNA methylation in the Igf2/H19 locus

Publication	Locus	Tissue	Change in methylation from PAE
Marjonen (2017)	H19 DMR (C6), H19 ICR	Placenta	Hypomethylation (nominal P-value only)
Masemola	H19 ICR (C6)	Blood, buccal	Hypomethylation
Marjonen (2018)	H19 ICR (C1 and C2), IGF2 DMR1	Placenta, embryo	No change
Haycock	H19 ICR (C1 and C2)	Placenta, embryo	Hypomethylation in placenta, no change in embryo
Zhou	H19 DMR, IGF2 DMR0, 1,2	Heart, brain, placenta	Hypomethylation at Igf2 DMR1, Igf2 DMR2, and H19, no change at Igf2 DMR0
Downing	IGF2 DMR1	Placenta, embryo	Hypomethylation
Stouder	H19 ICR	Sperm, brain, muscle, liver, tail	Hypomethylation in CTCF12 (F1) and all six sites (F2) in sperm, no change in other tissues (F1)

Among the imprinted genes, the *IGF2/H19* locus was the most consistently studied candidate region in eight different articles (3 human studies [13, 32, 38], 5 mouse models [33–37]). This transcriptional unit plays a central role in placental and embryonic growth. The locus contains several differentially methylated regions (DMRs) that are methylated in a parent of origin specific manner, and an imprinting control region (ICR) that modulates the transcription of *IGF2* and *H19* in an allele-specific manner [52].

Masemola et al. reported on four imprinted genes (*H19*-ICR, *IG*-DMR, *KvDMR1*, *PEG3*) of the genome in a South African cohort of FASD cases and controls in blood and buccal tissues [13]. Locus-wide hypomethylation was reported *PEG3* (average 7% lower in PAE cases), *KvDMR1* (effect size 1.49% lower in PAE cases) and IG-DMR1.B (average 0.84% lower in PAE cases), after adjusting for age and sex. No differences were observed at the *H19* ICR. The authors did not control for multiple testing in their analysis.

Marjonen et al. assessed DNA methylation at the *H19*-ICR and *H19*-DMR loci in placental tissue with high levels of PAE compared with and unexposed controls [32]. Overall, there was no difference observed between groups. Hypomethylation was reported at multiple CpG sites across the *H19* CTCF6 among carriers of the rs10732516 A/G genotype in the alcohol exposed group (effect size ~ 10–15% decrease), however this provided weak evidence for an association as it did not survive multiple testing correction.

Two mouse models of early exposure ranging from acute high to moderate dosages both examined effects on DNA methylation at *IGF2/H19* in embryos and placentas [33, 35]. Haycock et al. reported allele-specific hypomethylation on the paternal allele of H19-ICR in alcohol exposed placentas (effect size ~ 12–22% decrease) of C57Bl/6 & CastEi Hybrid Mouse but no difference in mid-gestation embryos [33]. Marjonen et al. did not report any significant differences in DNA methylation at the same regions in early embryos and placenta of C57Bl/6 mice with moderate PAE during the first trimester (10% ethanol w/v in drinking water, GD0.5–8.5), although they did not examine allele specific effects [35].

Zhou et al. reported hypomethylation at *Igf2* DMR locus in fetal heart (*Igf2*-*DMR1*), brain (*Igf2*-DMR2) and at the *H19* DMR locus in placental tissue from a C57Bl/6 mouse model of high dose exposure over the first 15 days of gestation (0.025 ethanol, GD0.5–15.5), which was twice the duration of the two previous studies. Effect sizes were 2–5% lower in the ethanol exposed fetal tissues. Tissue-specific effects of alcohol exposure were reported in this study, however allele specific effects were not examined. Downing et al. reported a small decrease in methylation at *IGF2*-DMR1 in embryonic tissue of C57Bl/6 mice following acute PAE (20% ethanol solution, GD0-8.5) but not in placental tissue (effect size ~ 7%) [34].

Stouder et al. examined trans-generation effects of low dose PAE in mid to late gestation (0.5 g/kg dam weight/day of ethanol, GD10-18) on eight week old offspring imprinted genes in normal FVB/N mice [37]. Hypomethylation at *H19*-ICR was reported in the sperm of F1 offspring, but not in somatic tissues (muscle, tail, brain, liver). Interestingly, this exposure-associated hypomethylation was not observed in F2 germ cells but rather in somatic brain tissues. Effect sizes ranged from 3–5% decrease in methylation after adjustment for multiple testing.

Meta-analysis of *IGF2*/*H19* regions was not feasible due to the lack of numerical reporting of methylation levels in some studies and differences in quantitative assays used to measure DNA methylation. Overall, there was limited but suggestive evidence for a reduction in DNA methylation at *IGF2*/*H19* regions in somatic tissues, but this was not entirely consistent, even among studies of the same tissue.

#### Methylome-wide association studies of PAE

There were four methylome-wide association studies (MWAS) assessing PAE, including two human studies using population based cohorts of PAE spanning birth to school age [53, 54] and two mouse models [16, 55]. All human studies employed the Infinium HumanMethylation450k array, and the two murine studies both employed methylated DNA immunoprecipitation (MeDIP). Tissues examined included whole blood, buccal swabs and cord blood, except for the mouse study which examined brain tissue.

Frey et al. conducted MWAS on buccal cell DNA from 156 primary school children with objectively measured PAE by ethyl glucuronide metabolite test (32 exposed, 124 unexposed) [53]. They report weak evidence of an association with PAE at 193 differentially CpGs (P < 0.001), however the effect was no longer evident after correcting for multiple tests.

Timms et al. conducted an MWAS on human cord blood samples from a UK based cohort of 253 newborns with PAE and 530 unexposed controls based on maternal self-report [54]. Their analysis reported on 192 genome-wide CpG sites with significant differences (false discovery rate < 0.05) associated with PAE.

Sharp et al. performed a meta-analysis from cord blood samples across six human cohorts (1147 PAE and 1928 non-PAE controls), and reported no significant differences in DNA methylation associated with PAE [20]. Confounding factors that were controlled for in all models included maternal age, educational attainment and smoking status in pregnancy. The control cohort was defined by PAE before and during the first trimester of pregnancy, compared to the case cohort that was defined by PAE both before and across pregnancy. Consequently, the study lacked a suitable control cohort characterised by no PAE. Technically this article meets our exclusion criteria, but we describe it here due to the scale and robustness of the study.

In a C57Bl/6 mouse model of voluntary maternal drinking (10% ethanol w/v in drinking water) in pregnancy (GD0-10), Laufer et al. used genome-wide analysis by Methylated DNA Immunoprecipitation (MeDIP-chip) of CpG islands in adult offspring whole brain DNA extracts [16]. The study found weak evidence for 6660 differentially methylated regions (un-adjusted P < 0.01) in alcohol-exposed mice. They also reported hypermethylation of imprinted regions *H19/Igf2,* as observed in placental tissue by Haycock [33], albeit in the opposite direction. There was no overlap in the aforementioned 3 MWAS studies of PAE with respect to CpG regions identified.

Alberry and Singh also adopted a C57Bl/6 mouse model with voluntary maternal drinking of 10% ethanol w/v in drinking water, but provided the ethanol throughout pregnancy and alongside plain drinking water. MeDIP sequencing was performed on the promoter regions of genes that are known to be associated with PAE or early life stress in hippocampal tissue from male offspring on postnatal day 70. The total number of genes that had promoters sequenced as part of the study is unclear, however the study identified 614 genes with hypomethylated promoters, 630 genes with hypermethylated promoters and 20 genes with both hypomethylated and hypermethylated promoters (unadjusted p < 0.01) before FDR correction. After FDR correction, no genes had promoters with a q-value below 0.01 which was defined as the statistical significance threshold in the study. If significance is defined as q < 0.05, three genes were identified as hypomethylated from PAE (*Krtap5-3* q = 0.03, *Hbegf* q = 0.03, *2310034C09Rik* q = 0.04) and one gene was hypermethylated from PAE (*Myo1a* q = 0.03) [55].

In summary, the PAE MWAS provide inconsistent evidence for any association between PAE and DNA methylation. Although each MWAS identified candidate DNA methylation loci that were significantly associated with PAE, there was a lack of replicated associations between studies.

#### Methylome-wide association studies of FASD

There have been four MWAS on children with FASD, which all employed the Infinium HumanMethylation450k array. Laufer et al. performed genome-wide methylation array analysis of buccal samples from 12 children (3–10 years) with FASD, and 11 age-matched controls of Northern European ancestry [56]. They report weak evidence of 269 differentially methylated regions (un-adjusted P < 0.005). Portales-Casamar et al. identified 658 CpG sites across the genome that were associated (FDR < 0.05) with FASD in a Canadian cohort of child and adolescent human buccal samples (110 FASD and 96 controls), including 41 CpG sites with a greater than 5% change in methylation [9]. Of the 658 CpGs identified as significant, 356 were hypermethylated in FASD cases and 302 were hypomethylated relative to controls (effect sizes 0.16–13.1%).

In a more recent study, Lussier et al. validated 161 of the 658 CpG sites associated with FASD in human buccal samples from an independent Canadian cohort of 48 children, reporting a similar direction of effect and statistically significant effect size [57]. Overall, the MWAS suggested moderate evidence for an association between DNA methylation and FASD with replicated associations reported in two of four studies. However, there was risk of bias arising from these studies where control groups were commonly children who had attended a clinic for a FASD diagnosis and not received a diagnosis [9, 56, 57] or the method for recruiting control groups was unclear [13, 58]. This suggests that control participants had developmental difficulties that may have been partly caused by epigenetic modifications and therefore did not represent a “typically developing” population. Secondly, a group of studies [9, 57] had imbalanced ethnicities between control and FASD groups (disproportionate number of First Nations children within case cohorts) and this may have affected their results even after they attempted to correct for this via statistical methods.

Okazaki et al. re-examined the cohorts from Lussier and Portales-Casamar studies to identify an association between FASD and five DNA methylation-based epigenetic clocks. The analysis was also performed on two peripheral blood cohorts, which included one Polish cohort and a second cohort from the Netherlands. The study identified one of the epigenetic clocks (GrimAge) was significantly associated with FASD in one buccal and both blood datasets, but no significant associations were reported with the other epigenetic clocks. Meta-analysis of datasets by tissue type also reported a significant association between FASD and the GrimAge epigenetic clock in peripheral blood, but no significant association was reported in buccal cells [59].

## Conclusions

Alcohol consumption in pregnancy has been linked to changes in offspring DNA methylation, which may be mechanistically important in mediating the harmful effects of alcohol on neurodevelopment. We have undertaken a systematic review of studies reporting associations between PAE and variations in DNA methylation. On balance, the results did not support a clear and consistent effect of PAE on DNA methylation, underscoring methodological limitations including substantial heterogeneity in study design, tissues examined, and methodologies used to assess DNA methylation, in addition to the risk of potential bias due to cellular heterogeneity within the tissues examined and inadequate control for confounding factors such as maternal diet and smoking, ancestry and cellular heterogeneity in tissues. Current knowledge regarding the epigenetic mechanisms of PAE remain unclear and findings from previous studies have not been reproducible to date. In contrast, studies examining the epigenetic changes from prenatal cigarette smoking have been found to be very reproducible across multiple cohorts [60, 61]. Ongoing cigarette smoking is likely to be more frequent than heavy alcohol use, which may explain the difference. If we consider the FASD phenotype to reflect heavy exposure, then some reproducible effects were observed although future studies will need to confirm this. Thus the supposition that PAE has no effect on offspring DNA methylation could not be ruled out. Results are reviewed in the context of these limitations with directions for future research.

Studies at imprinted regions, in particular, *IGF2*/*H19,* were the easiest to compare since methylation in this region is germline encoded and does not vary substantially across tissues and is less influenced by environmental exposures. We might reasonably expect that if alcohol were to have a strong effect on DNA methylation, substantial variation would be observed at these regions. On the contrary however, small hypomethylation differences were observed that were not consistent across studies with comparable exposure patterns. It is possible that compensatory mechanisms may exist, for example, the *ZFP57* gene encodes a transcriptional regulator that functions to maintain gene imprinting [62] in response to extracellular stimuli [63]. Similarly, studies that employed a marker of total nuclear DNA methylation content suggested that overall methylation levels were reasonably conserved. It is also possible that ethanol may have non-specific inhibitory regional effects on DNA methylation that would increase overall variability in methylation outcomes, even within the same tissue. If this were the case, we might expect this to be reflected in repeat elements of the genome or in global methylation assays. Two studies identified differential DNA methylation associated with FASD within the same loci [9, 57]. Such methylation changes might accumulate over time either as a consequence of FASD or through a causal mechanism, however further research is needed in other FASD populations. Given that the functional outcomes for those diagnosed with FASD can be completely different across a wide range of neurodevelopmental and physical domains, it could be hypothesised that the effects of PAE on DNA methylation are highly individualised. Potential contributing factors include the timing and dosage of alcohol exposure, characteristics of the parents including diet and health status, genetic factors, and other as yet unknown factors. Large individual differences could potentially be overlooked in the typical statistical approaches examining mean differences between groups. Therefore, personalised approaches looking for significant differences in DNA methylation between individuals prenatally exposed to alcohol compared and non-exposed populations might need to be considered.

In summary, there is a need for studies to employ more uniform designs and analysis plans including pre-defined criteria for assessing alcohol exposure and adjustment for a wider range of confounding factors including, but not necessarily limited to, mother’s age, diet (including folate and choline supplementation), child parity and gender, weeks of gestation, and ethnicity. Effect modifiers such as cell composition within tissues examined are equally important. These issues could be addressed by employing a matched study design or through adjustment during data analysis. For studies investigating FASD, samples were generally collected from 4 to 18 years after the exposure and birth. We know that children with FASD generally have additional life stressors compared with those who do not, such as increased adverse childhood experiences, and contact with the justice system (Lange et al., 2017). These in turn may have effects on epigenetic profiles within target tissues.

Other areas of study design were identified during the bias analysis that could generally be improved. A consistent issue in human studies was inclusion into the study criteria based on specimen availability. Given the stigma associated with alcohol use in pregnancy those who have used alcohol may be less likely to provide biological specimens. It is therefore imperative that analysis is undertaken to determine any potential bias this might introduce. Additionally, the questions used to ascertain PAE status were rarely specified, often involved retrospective collection and, as with all PAE studies, did not have use of an objective PAE biomarker. We additionally recommend more consistent reporting of methylation group mean differences to enable future meta-analyses. Finally, to address current limitations of cell heterogeneity in biospecimens such as blood and buccal, analysis of purified cell populations or single cell studies are likely to be informative in future research.

### Limitations of the review

This review focused exclusively on the association of offspring DNA methylation with maternal PAE. The advantage of including animal models in this review is that they are largely unaffected by confounding factors influencing DNA methylation in humans. Yet DNA methylation results from animal models may not be generalisable to humans due to differences between species. It is possible that some non-significant results were due to a lack of statistical power because of their small sample sizes and presence of residual confounding. Ensuring sample sizes have sufficient power to detect true effects is essential. However, several of the human studies that returned null results were of a reasonably large sample size. More research is warranted on DNA methylation from preconception alcohol exposure, including paternal alcohol use, and postnatal alcohol exposure transmitted through breastmilk, however these studies were outside the scope of this systematic review. Furthermore, studies such as those by Finegersh and Homanics suggest that paternal alcohol exposure can also affect the epigenetic outcomes for offspring. Whilst these studies were outside the scope of this review [64], this is an important area of future enquiry. Additionally, a meta-analysis could not be included in this review due to the differing tissues, species, sequencing methods and for targeted studies, loci, that were assessed between studies.

Prenatal alcohol exposure is a significant public health issue, and mechanistic understanding of the impact of this exposure on fetal development is urgently needed. This review summarises the current state of research and focus for future efforts to advance this area of science. Currently the evidence was inconsistent and inconclusive regarding maternal PAE as a critical exposure on offspring DNA methylation. Future studies in well characterized populations that focus on purified cells, or single cell analyses, will likely be informative.

## Supplementary Information

**Additional file 1: Fig. 1**. Individual study bias scores across all domains. Data are summarized as Figure 1 in the manuscript text.

## Data Availability

Not applicable.
