# Supplementary figures and images for: Association of prenatal alcohol exposure with offspring DNA methylation in mammals: a systematic review of the evidence
Source: Clin Epigenetics. 2022 Jan 21;14:12. doi: 10.1186/s13148-022-01231-9 (PMC8785586; doi:10.1186/s13148-022-01231-9)

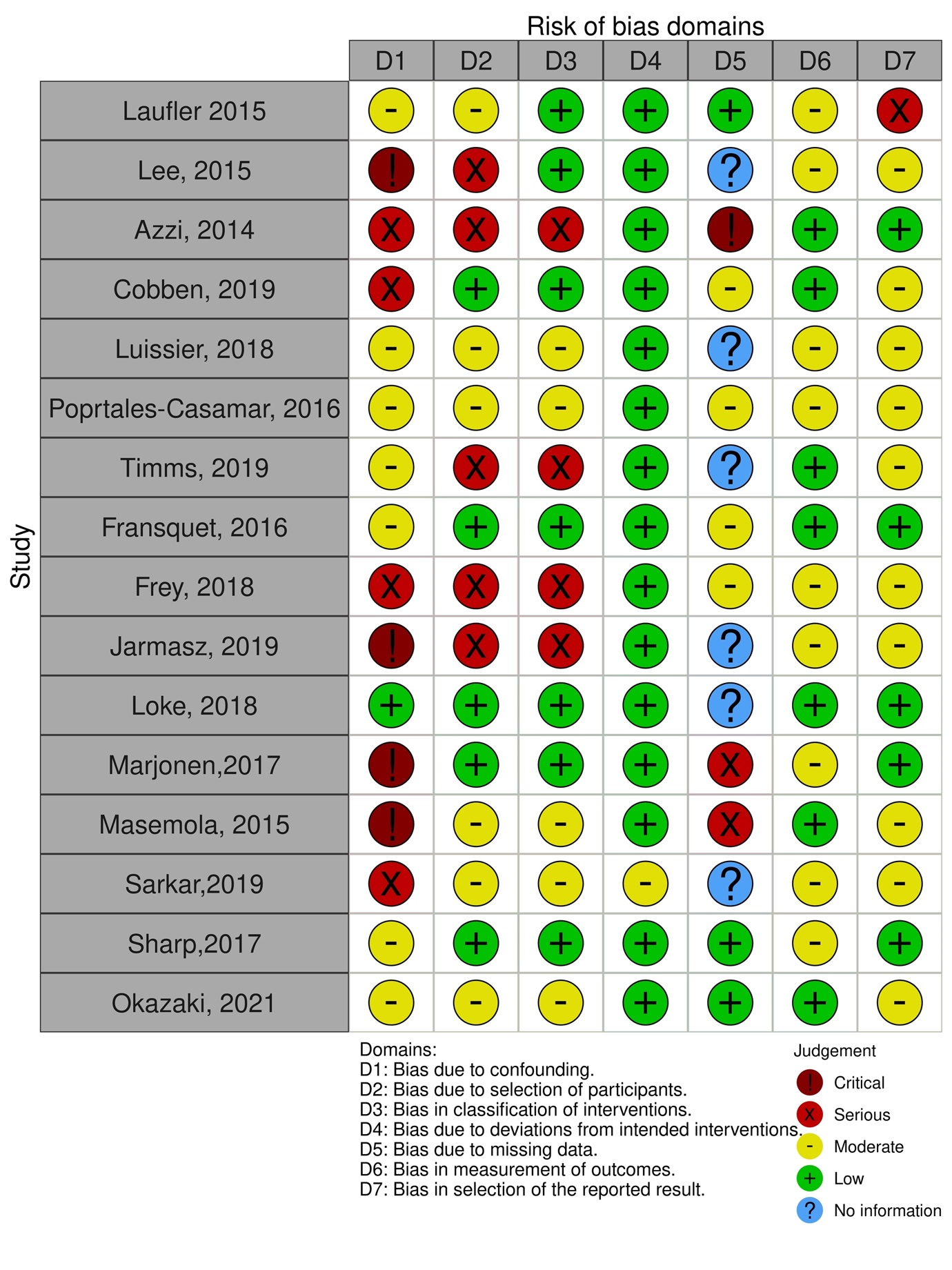

Supplement: Supplementary file 1 — Additional file 1: Fig. 1. Individual study bias scores across all domains. Data are summarized as Figure 1 in the manuscript text. [file 13148_2022_1231_MOESM1_ESM.png]
